# What Can Dietary Patterns Tell Us about the Nutrition Transition and Environmental Sustainability of Diets in Uganda?

**DOI:** 10.3390/nu11020342

**Published:** 2019-02-05

**Authors:** Carolyn Imelda Auma, Rebecca Pradeilles, Megan K. Blake, Michelle Holdsworth

**Affiliations:** 1 Public Health Section, School of Health and Related Research (ScHARR), University of Sheffield, Sheffield S1 4DA, UK; r.pradeilles@sheffield.ac.uk (R.P.); michelle.holdsworth@sheffield.ac.uk (M.H.); 2 Department of Geography, University of Sheffield, Sheffield S10 2TN, UK; m.blake@sheffield.ac.uk

**Keywords:** dietary patterns, nutrition transition, environmental sustainability, Uganda, women, rural, urban

## Abstract

Uganda is undergoing dietary transition, with possible environmental sustainability and health implications, particularly for women. To explore evidence for dietary transitions and identify how environmentally sustainable women’s dietary patterns are, principal component analysis was performed on dietary data collected using a 24 h recall during the Uganda Food Consumption Survey (*n* = 957). Four dietary patterns explained 23.6% of the variance. The “traditional, high-fat, medium environmental impact” pattern was characterized by high intakes of nuts/seeds, fats, oils and spreads, fish and boiled vegetables. High intakes of bread and buns, rice and pasta, tea and sugar characterized the “transitioning, processed, low environmental impact’ pattern. The ‘plant-based, low environmental impact” pattern was associated with high intakes of legumes, boiled roots/tubers, boiled traditional vegetables, fresh fruit and fried traditional cereals. High intakes of red/organ meats, chicken, and soups characterized the “animal-based high environmental impact” pattern. Urban residence was positively associated with “transitioning, processed, low environmental impact” (β = 1.19; 1.06, 1.32) and “animal-based high environmental impact” (β = 0.45; 0.28, 0.61) patterns; but negatively associated with the “plant-based low environmental impact” pattern (β= −0.49; −0.62, −0.37). A traditional, high-fat dietary pattern with medium environmental impact persists in both contexts. These findings provide some evidence that urban women’s diets are transitioning.

## 1. Introduction

Urbanization is rising globally, with estimates that by 2030 about 5 billion people, approximately 60% of the global population, will be urban dwellers [1,2,3]. The United Nations (UN) estimates that virtually all future urbanization will occur in low and middle-income countries (LMICs), such that by 2050 more than 50% of Africans will reside in urban areas [1,2]. Uganda is no exception as there are clear indications that the urban population is rapidly growing [4] and is estimated to have almost tripled by 2040 [5,6].

Urbanization has been associated with dietary changes commonly referred to as the “nutrition transition” [7,8,9,10]. The nutrition transition is characterized by a shift from traditional, less processed, plant-based diets, towards modern energy-dense, nutrient-poor diets characterized by high consumption of red and processed meats, sugar, fats and oils, refined carbohydrates and low fiber intake [11,12,13]. According to Popkin’s model, the early stages of the nutrition transition are marked by reduced consumption of starchy staples, coupled with increasing consumption of fruit, vegetables, and animal protein; gradually giving way to increased fat, refined carbohydrate, sugar, and processed food intake [11]. In the last stage of the nutrition transition, complex carbohydrate, healthier types of fat, fruit, and vegetable intake increase along with a reduction in the consumption of saturated fat, processed foods, and animal protein [11]. While all geographical areas in a country can undergo the nutrition transition, some authors have reported that at any one moment, urban and rural areas within the same country are likely to be at different stages of dietary change [8]. In sub-Saharan Africa (SSA), regardless of rural or urban context, women are disproportionately vulnerable to these dietary changes, more so if they are younger and of low-income [9,10]. The situation in Uganda concurs with this narrative, as Ugandan women of reproductive age (WRA) (15–49 years) have the poorest nutrition outcomes for all forms of malnutrition, compared with older women and men of the same age-group [6]. It follows, therefore, that these women may be more likely to be susceptible to the dietary changes concomitant with the nutrition transition. 

Some dietary changes associated with “transitioning diets” could be beneficial to nutritionally-vulnerable groups, e.g., increased iron and vitamin B12 intake from animal-source foods [12]. However, evidence from some middle and high-income countries shows that these dietary changes are associated with an increased risk for nutrition-related non-communicable diseases, e.g., some cancers, type 2 diabetes, and cardiovascular disease, when accompanied by increased sedentarism [13,14,15]. This is exemplified by China, a middle-income country, in which a recent increase in consumption of edible oils, animal protein, and refined carbohydrates in place of legumes and coarse grains, has been accompanied by an increase in obesity and non-communicable disease prevalence [16]. Additionally, from an environmental sustainability perspective, evidence from high-income countries (HICs), indicates that diets high in red and processed meats and low in fruit and vegetables, legumes, cereals, roots, and tubers, prevalent in many HICs, are associated with high greenhouse gas emissions (GHGEs), land-use change, and greater fresh water use, which could have negative consequences for global warming and consequently climate change [15,17,18,19]. It follows, therefore, that changing dietary patterns in increasingly urbanizing SSA countries could have negative implications for both health and environmental sustainability, depending on the magnitude of dietary change. 

The aim of this study, therefore, was to generate dietary patterns of rural and urban Ugandan women of reproductive age, and thereby: (i) Identify if there is evidence of dietary transitions using rural-urban comparisons as a proxy and (ii) assess the environmental sustainability of the identified dietary patterns. 

## 2. Materials and Methods

### 2.1. Survey Design and Sampling

Data were obtained from the Uganda Food Consumption Survey (UFCS) [20,21]. The UFCS, a cross-sectional survey conducted between May and September 2008. It remains the most recent nationally representative food consumption survey in Uganda, even though there have been subsequent regional-level surveys. The UFCS was undertaken in three regions, collectively data representing rural and urban Uganda. A representative sample of women (*n* = 957) aged 15–49 years was recruited using a multi-stage cluster sampling technique. The sampling procedure used for the UFCS has been published in full detail elsewhere [20,21]. 

Ethical approval for the 2008 UFCS was obtained from the Ugandan National Council for Science and Technology (UNCST) on 30 April 2008 and from the AED/Research Integrity Department in Washington, DC on 5 January 2008 [20,21]. The University of Sheffield, School of Health and Related Research (ScHARR) Ethics Committee granted ethical approval for the use and analysis of the secondary dietary data in October 2016.

### 2.2. Data Collection

A single quantitative 24 h recall was used to collect information on all the food and drink consumed by participants in the 24 h preceding the interview. To ensure accuracy of the data, the multi-pass method was employed [20,21]. Locally-developed food picture charts were used to estimate portion sizes for dietary intake during the last stage of the multi-pass interview [20,21]. Dietary data were collected by trained field assistants who were primarily graduates of either Human Nutrition or Food Science and Technology from Makerere University [20]. Interviews took place at participants’ households on all days of the week to account for variations between weekday and weekend day intakes [20,21]. Data were not collected on “festive” days since on such day’s intakes are not representative of usual intakes [21]. Demographic data of participants, i.e., age, occupation, level of education, socioeconomic status, and marital status were collected [20,21] but were unavailable for use at the time of this study. 

### 2.3. Data Management and Statistical Analyses

Dietary patterns were generated using Principal Component Analysis (PCA). The number of food items entered as input variables in a PCA depends on the sample size, and consequently may impact on the results obtained. Given the sample size available at analysis (*n* = 955), the 531 food items reported in the UFCS were recoded into 35 smaller categories to allow a robust analysis (Table S1). The classification of these 35 food categories was based on: Culinary use, whether the food item was described as part of the traditional Ugandan diet in the literature [22,23,24,25,26,27,28]; and current evidence around environmental impact in terms of GHGEs (kgCO2eq per kg) [29,30,31,32] and nutritional content from food composition tables [33,34]. To this end, the 531 food items were first placed in larger food groups based on similarities in nutrient content, after which these larger groups were refined into 35 smaller food categories based on culinary use in the local context and whether items were “traditional” Ugandan foods or “modern”. Lastly, the 35 food categories were categorized as low, medium or high impact (Table S1) depending on whether the food items that they were primarily composed of were defined as low, medium or high impact (Table S2). Foods items were categorized as low impact if they had GHGEs <4 kgCO2eq per kg of product, medium impact if they had GHGEs between 4–7 kgCO2eq per kg of product while those >7 kgCO2eq per kg of product were classified as high impact foods [29,32] (Table S2). 

A participant’s intake (grams) for each of the 35 food categories was obtained by summing their food intake (grams) over the 24 h period for any of the food items in each respective food category. Food intake (grams) for the food categories was then standardized to account for portion size effect [34]. Standardized intakes were used to identify possible outliers (±6SD) [35]. For individuals with intakes identified as possible outliers, food intake (grams) for all food items consumed over the 24 h period was scrutinized for plausibility, based on knowledge of the local context. Intakes deemed biologically implausible were removed from the dataset, but not the individual’s entire dietary intake. 

PCA was run with the standardized intakes as the input variables, with missing cases excluded pairwise, to account for implausible intakes removed during data cleaning. Varimax rotation, with Kaiser Normalization, was used to interpret the components [36]. Eigenvalues >1, in combination with the scree plot (inflection point), were used to identify possible components to retain. The final decision to retain components was then based on both statistical information (i.e., scree plot and eigenvalues) and interpretability of the components [36]. In interpreting and labeling dietary patterns, food items with factor loadings of ≥±0.2 were considered to load either highly positively or negatively on each dietary pattern [36,37,38,39]. The final labeling of dietary patterns was based on the qualities of the food categories that characterized the principal component. Food categories that loaded highly positively or negatively on each dietary pattern were identified using the ±0.2 threshold. For each principal component, a count of the total number of positive and negative loading food categories at each environmental impact level (low, medium, high) was obtained (Table S3). The difference between the total number of positive and negative counts at each environmental impact level was then obtained for each dietary pattern. The environmental impact category (low, medium, high) with the largest difference of the three was taken as the environmental impact category representative of that dietary pattern, and it was therefore labelled to reflect this (Table S3). When a principal component had an equal difference for more than one environmental impact category, the final decision on labelling depended on the environmental impact category of the food category that had the highest positive factor loading on that dietary pattern. In addition to environmental impact categories, dietary patterns were also labelled depending on whether the food categories constituting them could be considered as modern or traditional. This approach of labelling dietary patterns based on several criteria has generally been used by authors who have used PCA.

To explore the association between urban/rural residence and the retained dietary patterns, bootstrap linear regression analysis was performed. Bootstrap linear regression is a robust method appropriate for use when not all the assumptions for a linear regression analysis are met [40]. All data management and analysis were performed using Statistical Program for the Social Sciences (SPSS) Version 23. 

## 3. Results

### 3.1. Sample Characteristics

Although 957 women were sampled for the UFCS, dietary data of only 955 women were available for use at the time of this study. The sample of women who participated in the UFCS was largely rural (67.2%) compared with urban (32.8%) [20,21]. Most study participants were younger than 35 years of age and were either married or living with a partner. Urban women were more highly educated than their rural counterparts, with 90% of them having completed at least the primary level of schooling, compared with 72% of rural participants. Urban participants were also relatively wealthier than rural participants. Over 75% of urban participants were in the two highest wealth quintiles, while approximately 65% of rural participants occupied the two lowest wealth quintiles [20,21]. Although rural participants were primarily involved in agricultural activities, urban participants were involved in more formal employment, i.e., trade and the service industry. 

Details of the socioeconomic and demographic characteristics of the study participants from the UFCS are described in full elsewhere [20,21].

### 3.2. Characteristics of the Dietary Patterns

A considerable number of principal components had eigenvalues >1.0, however, only the first four components were retained based on the scree-plot and interpretability [37]. These four distinct dietary patterns collectively explained as 23.6% of the variance in food intake among this sample of Ugandan WRA (Table 1). The first dietary pattern explained 7.6% of the variance and had only high positive factor loadings for fish, traditional and non-traditional fats, oils and spreads, nuts and seeds, boiled traditional vegetables, and boiled traditional cereals. This pattern was labelled the “traditional, high-fat, medium environmental impact” dietary pattern (Table 1). 

The second dietary pattern explained 6.0% of the variance in food intake, and was characterized by high intake of tea, sugar, bread and buns, rice and pasta, and low intake of boiled traditional vegetables and boiled traditional cereals (Table 1). This pattern was composed of food categories not cited in the literature as part of the traditional Ugandan diet. This dietary pattern had some characteristics of a “modern diet” and was therefore labelled the “transitioning, processed, low environmental impact” dietary pattern. The third dietary pattern explained 5.4% of the variance and was associated with high consumption of fresh fruit, boiled non-traditional vegetables, boiled roots and tubers, fried traditional cereals and legumes, and low consumption of boiled traditional cereals (Table 1). This pattern reflected a mixture of plant-based traditional and non-traditional food items and was therefore labelled the “plant-based, low environmental impact” dietary pattern. The fourth dietary pattern accounted for 4.6% of the variance and was characterized by high consumption of red and organ meat, chicken and soups, and low intake of legumes (Table 1). The pattern was labelled “animal-based, high environmental impact” dietary pattern since it was not associated with any low-impact plant-based food groups.

### 3.3. Dietary Patterns and Place of Residence

While the traditional, high-fat dietary pattern with medium environmental impact appears to be consumed by both rural and urban Ugandan women, linear regression results suggest that urban residency was positively associated with both the “transitioning, processed, low environmental impact” (β = 1.19; 95%CI [1.06, 1.32]) and the “animal-based, high environmental impact” (β = 0.45; 95%CI [0.28, 0.61]) dietary patterns (Table 2). On the other hand, urban residency was negatively associated (β = −0.49; 95%CI [−0.62, −0.37]) with the more environmentally sustainable “plant-based, low environmental impact” dietary pattern. 

## 4. Discussion

The aim of this study was to identify how environmentally sustainable Ugandan women’s dietary patterns are and explore evidence for dietary transitions using rural-urban comparisons. In this cross-sectional study, four dietary patterns, explaining 23.6% of the variance in food intake among participants, were identified: “Traditional, high-fat, medium environmental impact”; “transitioning, processed, low environmental impact”; “plant-based, low environmental impact” and “animal-based, high environmental impact”. The “transitioning, processed, low environmental impact” and “animal-based, high environmental impact” dietary patterns were positively associated with urban residency, while the “plant-based, low environmental impact” was negatively associated with urban residency. 

These findings are consistent with a Ghanaian study, in which three dietary patterns explained 29% of the variance in dietary intake among rural, urban, and migrant adults [41]. However, among rural Tanzanian women and South African adults, a larger number of dietary patterns explained more than 40% of variance in food consumption [38,42]. Some authors have proposed that the number of dietary patterns, and consequently the variance that can be explained by each dietary pattern, depends on the number of food groups used in the PCA [43]. The fact that 35 food categories were used in this study could explain why the total variance accounted for by the four dietary patterns was <30%; compared with the 17 food groups in South Africa [38] and 12 food groups in Tanzania [42]. However, in order to represent the heterogeneity of such a multi-ethnic research setting, it was necessary to include a relatively large number of food groups. Moreover, a similar number of food categories have been used to reflect the diversity of food intake in a recent study exploring dietary patterns in four African settings [44]. 

The “traditional, high-fat, medium environmental impact” pattern found in our study, although largely traditional, could be indicative of early stages of the nutrition transition, which is typically characterized by increased consumption of inexpensive fats and oils alongside the traditional diet [45]. This dietary pattern shows some similarities to the “roots, tubers and plantain” dietary pattern found in Ghana. While both dietary patterns were characterized by high consumption of traditional cereals, nuts and seeds, and fats and oils, the main difference between them was the high factor loading of roots, tubers and plantain, refined cereals and fruits on the “roots, tubers and plantain” dietary pattern [41], which was contrary to the “traditional, high fat, medium impact” dietary pattern in our study. 

The “transitioning, processed, low environmental impact” dietary pattern, characterized by a high intake of bread and buns, rice and pasta, sugar and sweeteners and tea, provides evidence of dietary transition, particularly in urban Uganda. This dietary pattern differed from the “snacking” pattern, a transitioning dietary pattern, identified among adults (20-65 years) in Burkina Faso [46] and the “rice, pasta, meat and fish” dietary pattern among adults in Benin [35]. While both dietary patterns were associated with urban residency and had high intake of sugar, the “snacking” pattern, was also associated with a high consumption of fried foods, vegetable fat, cereals, sweetened drinks, vegetables, dairy products, fresh fish, roots and tubers, lean meats and poultry. This dietary pattern was also somewhat similar to the “processed” dietary pattern identified among urban, rural and peri-urban South African, Tanzanian and Ugandan women [44]. However, the main difference was that the “processed” dietary pattern was also characterized by high consumption of processed animal products [44]. This could be attributed in part to a largely urban and relatively highly-educated sample in their study, relative to ours. Given the fairly large rural sample in our study, it is unknown whether food taboos that discourage meat consumption among women might have played a role in our study. 

On the other hand, our “transitioning, processed, low environmental impact” was very similar to the “purchase” pattern among rural Tanzanian women, characterized by a high consumption of bread or cakes, tea and sugar [42]. These similarities suggest that urban Ugandan women and rural Tanzanian women could be at a similar early-mid stage of the nutrition transition, which corroborate recent reports that Uganda is in the early-mid stages of the nutrition transition [47]. Although our findings do not exactly mirror the transitioning diet narrative common in some LMICs, the manifestation of some elements of the so-called ‘*Westernized*’ diet, alongside traditional dietary attributes in this study, can be explained in part by authors who argue that such mixed dietary patterns have been found in SSA countries at similar stages of the nutrition transition, moreover dietary change is a gradual process in which people often retain attributes of their traditional practices alongside new ones [42,46,48]. Accordingly, high consumption of meat, dairy products and energy-dense, nutrient-poor foods, common in many HICS, are characteristically found in later stages of the nutrition transition [42]. 

The “plant-based, low environmental impact” pattern that emerged was characterized by a diverse mix of plant-based food groups, i.e., fruits, vegetables, legumes, roots and tubers and cereals, which more recently, have been highlighted as the cornerstone for both health and environmental sustainability owing to their lower GHGEs, particularly when they are field-grown [29,30,31,32,49], as is the case in Uganda. On the other hand, the “animal-based, high environmental impact” dietary pattern was largely animal-based. This dietary pattern was similar to the “animal-based” pattern among urban male and female South African adults [38]; the “modernity” pattern, among adults in Burkina Faso [46]; and the “animal products” pattern among rural Tanzanian women [42]. Although the “plant-based, low environmental impact” pattern was entirely plant-based, it is important to note that all other dietary patterns, with the exception of the “animal-based, high environmental impact” pattern also had a high proportion of low and medium-impact plant-based food categories, implying that the Ugandan diet remains predominantly plant-based, as has been previously highlighted [20].

The differences between dietary patterns in our study and those in other SSA countries could be attributed to the strong influence of the geographical and cultural environment on dietary practices, however, it must also be noted that dietary data in these other studies included different sub-groups than ours. For example, in some instances dietary data from older participants were included [46]; while in other studies dietary intakes of both male and female participants were considered [35,38,39,41,46]. It may be argued that including data from male participants in some of the studies could account to some extent for the differences in dietary patterns between their studies and ours, particularly where meat consumption is concerned. While on the one hand, a 2005 study cited the notion that men consider meat an indicator of their masculinity [50], the finding that no differences existed in dietary patterns between adult males and females in two studies [44,46] suggest that of the exclusion of data from men in our study may not have had a strong bearing on the resulting dietary patterns. 

Some strengths and limitations of our study must be highlighted. To the best of our knowledge, this is the first study that describes dietary patterns of Ugandan WRA, through both a transitioning and environmental sustainability lens. Data for this study were drawn from a large representative sample (*n* = 955) that included participants across a range of socio-economic, occupation, and ethnic groups, possibly increasing the generalizability of these findings to other Ugandan WRA. Our sample was disproportionately rural, which can be highlighted as a limitation. However, we believe our sample was somewhat reflective of the skewed population distribution in the country given that approximately 7 in 10 WRA live in rural areas [4] while about 80% of the general population are rural dwellers [4,5]. On the other hand, PCA has been criticized on many fronts, stemming from the potential subjectivity in the analytical process, right from the creation of food groups, to the retention, labeling, and interpretation of principal components, limitations we acknowledge. To this end, some authors argue that dietary patterns obtained from data-driven approaches lack stability over time [35]. Moreover, our study was cross-sectional in nature, using data collected in 2008. This means associations between urbanization and dietary changes could not be studied prospectively. Additionally, dietary patterns among urban and rural Ugandan WRA could have changed between then and now. While we acknowledge this as one of our study’s limitations, it was pertinent to fill the dearth of information on population-level dietary patterns derived using such methods in Uganda, and indeed SSA. More recent surveys have been carried out since the UFCS, e.g., the nationally-representative 2014 Uganda non-communicable disease (NCDs) risk factors STEPS survey, which collected data on men and women’s fruit and vegetable consumption patterns and the Africa/Harvard School of Public Health Partnership for Cohort Research and Training (PaCT) pilot study, which collected food consumption data from rural and peri-urban men and women using an FFQ. However, at the time of analysis, the 2008 UFCS was the most comprehensive dietary intake survey available, collected from a nationally-representative sample of both rural and urban Ugandan women. We were also unable to carry out estimations for energy intake in our study, which is a limitation that we acknowledge. Lastly, data for this study were collected using a single 24 h recall. Although the 24 h recall is the most appropriate for use in resource-limited contexts where populations might be unable to keep food records, it is limited by recall bias and relies heavily on the skills of the interviewer. However, for the UFCS, data were collected by trained interviewers. Additionally, in each of the sampled regions, repeat 24 h recall interviews were conducted on 10% of the study participants on a non-consecutive day to account for intra-individual variation in food intake [20,21].

## 5. Conclusions

While the Ugandan diet remains largely based around low and medium-impact plant-based foods, compared with rural women, urban women consumed a more varied diet that incorporated more animal products. The first two dietary patterns responsible for the most variability in dietary intake indicated that this sample of urban Ugandan women is at mid stage of the nutrition transition. The high-fat intake amidst traditional dietary practices, could also point to early stages in the nutrition transition among rural Ugandan women. The ‘Western dietary pattern’, with which our transitioning dietary patterns bear some similarity, has been associated with increased risk for obesity and nutrition-related NCDs. More recently the ’Western dietary pattern’ has been associated with an increased risk of high-risk Human Papillomavirus (hrHPV) infection and cervical cancer [51], one of the leading causes of cancer mortality among Ugandan women [52]. The findings from our study could therefore allude to a subsequent increased risk for such diseases among both rural and urban Ugandan women, which could have dire consequences given the country’s already fragile public health system.

Future research will need to identify the factors influencing dietary transitions in both rural and urban contexts. Public health practitioners and policy makers will have to consider the varied dietary landscape in Uganda in designing recommendations, policies, and interventions for healthy and environmentally sustainable diets and dietary practices. 

## Figures and Tables

**Table 1:** Factor loadings of the 35 food categories on the four dietary patterns.

Dietary Patterns	Traditional, High-Fat, Medium Environmental Impact	Transitioning, Processed, Low Environmental Impact	Plant-Based, Low Environmental Impact	Animal-Based, High Environmental Impact
**Food Categories**				
Red meat	−0.01	0.18	−0.05	**0.73**
Organ meats	−0.07	−0.07	0.02	**0.60**
Chicken	−0.01	−0.06	0.13	**0.21**
Fish	**0.71**	0.16	−0.06	0.07
Insects	0.01	0.01	−0.13	−0.10
Sugar	0.14	**0.56**	0.13	0.04
Bread and Buns	0.11	**0.66**	−0.15	0.14
Chapatti	0.05	0.12	−0.14	−0.03
Eggs	0.01	0.15	0.05	0.03
Rice and pasta	−0.03	**0.55**	−0.05	0.08
Traditional fats, oils and spreads	**0.29**	**−0.20**	−0.05	−0.00
Non-traditional fats, oils and spreads	**0.75**	0.07	0.01	−0.05
Milk	0.04	0.09	0.02	0.05
Alcoholic drinks	0.03	−0.07	−0.04	−0.01
Porridge	−0.02	−0.01	−0.06	0.05
Savoury Snacks	−0.03	0.03	−0.02	0.17
Sugary Drinks	−0.04	−0.06	−0.06	0.02
Soups	0.02	0.11	−0.02	**0.78**
Tea	**−0.37**	**0.53**	0.17	0.08
Nuts and seeds	**0.30**	−0.13	−0.05	−0.02
Groundnut sauce	−0.10	0.16	−0.03	−0.09
Fast food	0.05	−0.05	0.04	−0.11
Fresh fruit	0.09	0.13	**0.66**	0.01
Traditional vegetables (boiled)	**0.27**	**−0.33**	−0.18	−0.11
Traditional vegetables (fried)	0.00	−0.08	−0.06	−0.02
Non-traditional vegetables (fried)	0.08	−0.03	0.08	−0.01
Non-traditional vegetables (boiled)	**0.49**	0.02	**0.39**	−0.07
Roots and tubers (boiled)	−0.14	−0.10	**0.64**	−0.06
Roots and tubers (fried)	0.00	0.05	0.01	−0.01
Katogo	−0.07	0.01	0.12	−0.06
Traditional cereals (boiled)	**0.41**	**−0.23**	**−0.29**	−0.05
Traditional cereals (fried)	−0.05	−0.16	**0.54**	0.02
Legumes	−0.07	0.02	**0.43**	**−0.22**
Matooke	−0.13	−0.06	−0.05	0.163
Sweets	0.05	0.11	−0.10	−0.02
Variance (%)	7.65	5.97	5.37	4.57
Total variance (%)	23.56			

High positive or high negative factor loadings on dietary patterns are highlighted in bold.

**Table 2:** Bootstrap linear regression results for associations between retained dietary patterns and urban/rural residence among Ugandan women of reproductive Age.

Dietary Pattern	β-Coefficient	95% CI	*p*-Value
**1**	0.10	(−0.04, 0.22)	0.189
**2**	1.19	(1.06, 1.32)	0.001 **
**3**	−0.49	(−0.62, −0.37)	0.001 **
**4**	0.45	(0.28, 0.61)	0.001 **

*p*-values with ** are statistically significant (*p* < 0.01).

## Supplementary Materials

The following are available online at http://www.mdpi.com/2072-6643/11/2/342/s1, Table S1: Food groups used in the generation of dietary patterns for Ugandan women of reproductive age. Table S2: Environmental impact categories for food groups used in the PCA of Ugandan women’s diets. Table S3: Counts of food categories loading positively and negatively on dietary patterns and resulting labels.
