# Supplementary material for: What Can Dietary Patterns Tell Us about the Nutrition Transition and Environmental Sustainability of Diets in Uganda?
Source: Nutrients. 2019 Feb 5;11(2):342. doi: 10.3390/nu11020342 (PMC6412986; doi:10.3390/nu11020342)
Supplement: Supplementary file 1 [file nutrients-11-00342-s001.pdf]

Table S1: Food Groups used in the Generation of Dietary Patterns for Ugandan Women of Reproductive Age

Food Group	Example food items from the UFCS 24hr recall	Environmental Impact Category
Roots and tubers (boiled)	Yam, sweet potato, ('irish') potato, cocoyam, cassava, cassava: maize 'atap', cassava 'atap' (cooked by boiling/steaming/eaten raw)	low
Roots and tubers (fried)	Sweet potato, cassava (cooked by shallow/deep-fat frying)	low
Matooke	Matooke (cooking bananas) (cooked by steaming or boiling)	low
Traditional cereals (boiled)	Maize, millet, sorghum, sorghum: cassava (2:1) 'atap', (cooked by boiling/steaming)	low
Traditional cereals (fried)	Popcorn, deep-fried maize (hard corn), roasted maize	low
Rice and pasta	White and brown rice, spaghetti, macaroni	medium
Bread and Buns	Sweet/salty brown/white bread, buns	low
Chapatti	Chapatti	low
Red meat	Beef, Goat meat, Pork	high
Chicken	Chicken	medium
Fish	Nile tilapia, Nile perch, 'mukene', 'nkejje', 'semutundu' (fresh, smoked or sun-dried)	medium
Organ meats	Intestines (cow/goat), tongue, heart, kidneys, chicken gizzards, 'mulokonyi' (cow hooves)	high
Insects	Grasshoppers, white ants (termites), bee larvae	low
Milk and milk products	Milk (boiled and fresh), yoghurt, milk tea, 'eshabwe', sour milk	medium
Eggs	Boiled eggs, fried egg	medium
Legumes	Beans ('nambaale'/ yellow/ white/ black/ kidney/ adzuki) with/without skin; bean sauce; field pea sauce; cow pea sauce; pigeon pea sauce	low
Nuts and seeds	Pumpkin seeds, sunflower seeds, roasted groundnut seeds, roasted sesame seeds, etc.	low
Groundnut sauce	Groundnut sauce (sauce made from raw milled ground groundnuts and water), raw pounded groundnuts, sesame seed sauce	low
Fresh fruit and unsweetened juice	Orange, pineapple, mangoes, guavas, raspberries, 'nsali', banana, passion fruit, papaya, guava, lemon, jackfruit, 'empafu'(olive), tangerine, watermelon, fresh fruit juice with no sugar added	low
Traditional vegetables (boiled)	'nakati', spider plant, 'dodo', pumpkin leaves, 'malakwang', cow pea leaves, bean leaves, ntula, okra fruit, okra leaves, pumpkin, 'ensusuti', alayo. (cooked by boiling/steaming/eaten raw)	low

Traditional vegetables (fried)	'nakati', spider plant, 'dodo', pumpkin leaves, 'malakwang', cow pea leaves, bean leaves, ntula, okra fruit, okra leaves, pumpkin, 'ensusuti', alayo (cooked by frying with oil)	low
Non-traditional vegetables boiled	Tomatoes, chilli, cucumber, onions, garlic, avocado, carrots, green bell peppers, 'Sukuma wiki' (kale), French beans (green beans), cabbage (cooked by boiling/steaming/eaten raw)	low
Non-traditional vegetables fried	Tomatoes, chilli, cucumber, onions, garlic, avocado, carrots, green bell peppers, 'Sukuma wiki' (kale), French beans (green beans), cabbage (cooked by frying with oil)	low
Traditional fats, oils and spreads	Shea nut butter, 'Muzigo Muganda' (cow ghee), groundnut paste, sesame paste, groundnut-sesame paste	medium
Non-traditional fats, oils and spreads	Vegetable oil, margarine, hydrogenated vegetable fat	medium
Katogo	Various combinations of yam, matooke, potatoes, sweet potatoes, legumes, and vegetables in one dish; 'nyoyo'	low
Porridge	Porridges (from flours of any combination traditional/non-traditional cereals or from composite tubers/roots and cereals flours), 'enturire'	low
Fast food	Chips, chaps, chips and liver, sausages, rolex, etc	medium
Soups	Soups (from beans, beef, goat meat, chicken, pork or fish), tomato soup	low
Sweets	Biscuits, candies, 'kabalagala', gulusa, doughnuts, 'mandazi', half cake, cake	medium
Savoury snacks	Samosas, rice balls	low
Sugar	Cane sugar (white and brown), sugarcane, honey	low
Sugar-sweetened beverages	Fruit juice with sugar added, soda (carbonated drinks), 'safi', 'splash' (fruit-flavoured drinks and concentrates)	low
Tea	Tea	low
Alcohol	Processed (bottled) beer, processed (bottled) wine, traditional beers, 'mwenge bigere', 'ajono', 'tonto', 'malwa' (crude beers and gin)	low

Table S2. Environmental Impact Categories for Food Groups used in the PCA of Ugandan Women's Diets*^.

low impact (< 1.0 kg CO2 e/kg)	medium impact (1.0-4.0 kg CO2 e/kg)	high impact (>4.0 kg CO2 e/kg)
traditional vegetables boiled	milk and milk products	red meat (pork, goat and beef)
traditional vegetables fried	chicken	organ meats
non-traditional vegetables boiled	fish	
non-traditional vegetables fried	rice and pasta	
tubers and roots boiled	eggs	
tubers and roots fried	fast food	

traditional cereals boiled	traditional fats, oils and spreads	
traditional cereals fried	non-traditional fats, oils and spreads	
legumes	sweets	
bread and buns	savoury snacks	
sugar		
groundnut sauce		
fruits		
soups		
insects		
porridge (derived from cereals, roots and tubers)		
nuts and seeds		
chapatti		
sugary drinks		
alcohol		
katogo (plant-based mixed dish)		
matooke (cooking bananas - plantain)		

*Macdiarmid et al. 2012, ^ Clune et al. 2017.

Table S3.Counts of food categories loading +vely and -vely on dietary patterns and resulting labels

	Count of food categories loading +vely and -vely on principal components						Difference in counts			
principal component (PC)	low impact (count for +ve factor loading)	low impact (count for -ve factor loading)	medium impact (count of +ve factor loading)	medium impact (count of -ve factor loading)	high impact (count for +ve factor loading)	high impact (count for -ve factor loading)	low	medium	high	label ascribed to dietary pattern
PC1	3	1	4	0	0	0	2	4	0	medium
PC2	3	2	1	1	0	0	1	0	0	low
PC3	5	1	0	0	0	0	4	0	0	low
PC4	1	1	1	0	3	0	0	1	3	high

*principal components correspond with dietary patterns

NOTES

1. Using thresholds from Mcdiarmid et al. (2012) food groups were classified as low, medium or high depending
foods in the dataset undergo minimal food processing and the most energy-intensive stage occurs at the primary p
Additionally, most food waste occurs post-harvest. It was therefore it was assumed that the highest environmenta
consumption stage i.e. when produce is still fresh for most foods. For this reason, the thresholds were applied to th
which were obtained from the systematic review by Clune et al. (2017). For a few processed foods e.g. cakes, infor
al. (2012).

2. For insects, whose information was not in either papers, information about impact was obtained from:

(i) Oonincx, D. G. A. B.; de Boer, I. J. M. Environmental Impact of the Production of Mealworms as a Protein Sourc
2012, *7*, 1-5, doi: 10.1371/journal.pone.0051145

(ii) Oonincx DGAB, van Itterbeeck J, Heetkamp MJW, van den Brand H, van Loon JJA, et al. (2010) An Exploratior
Insect Species Suitable for Animal or Human Consumption. PLoS One, **2010**, *5*, 1-7, doi: 10.1371/journal.pone.0C
